# Underestimation of mitochondrial respiratory capacity in gray matter voxels of the human brain map due to limited OXPHOS and TCA cycle in astrocytes

**DOI:** 10.3389/fncel.2025.1661231

**Published:** 2025-08-13

**Authors:** Christos Chinopoulos

**Affiliations:** Department of Biochemistry, Semmelweis University, Budapest, Hungary

**Keywords:** mitochondria, astrocytes, MRI, OXPHOS, respiratory capacity, neurons

## Abstract

Considering that the aerobic energetic landscape of the brain is shaped by its mitochondria, Mosharov et al. generated an atlas of mitochondrial content and enzymatic OXPHOS activities at a resolution comparable to MRI by physically voxelizing frozen human brain tissue. However, astrocytes in the adult human brain lack expression of several TCA cycle and OXPHOS enzymes. Therefore, their formula expressing mitochondrial respiratory capacity (MRC) -defined as tissue respiratory capacity normalized to mitochondrial density- underestimates actual values by a factor proportional to the square root of the fraction of respiration-capable cells (primarily neurons) in gray matter voxels.

## Introduction

**Figure 1:**
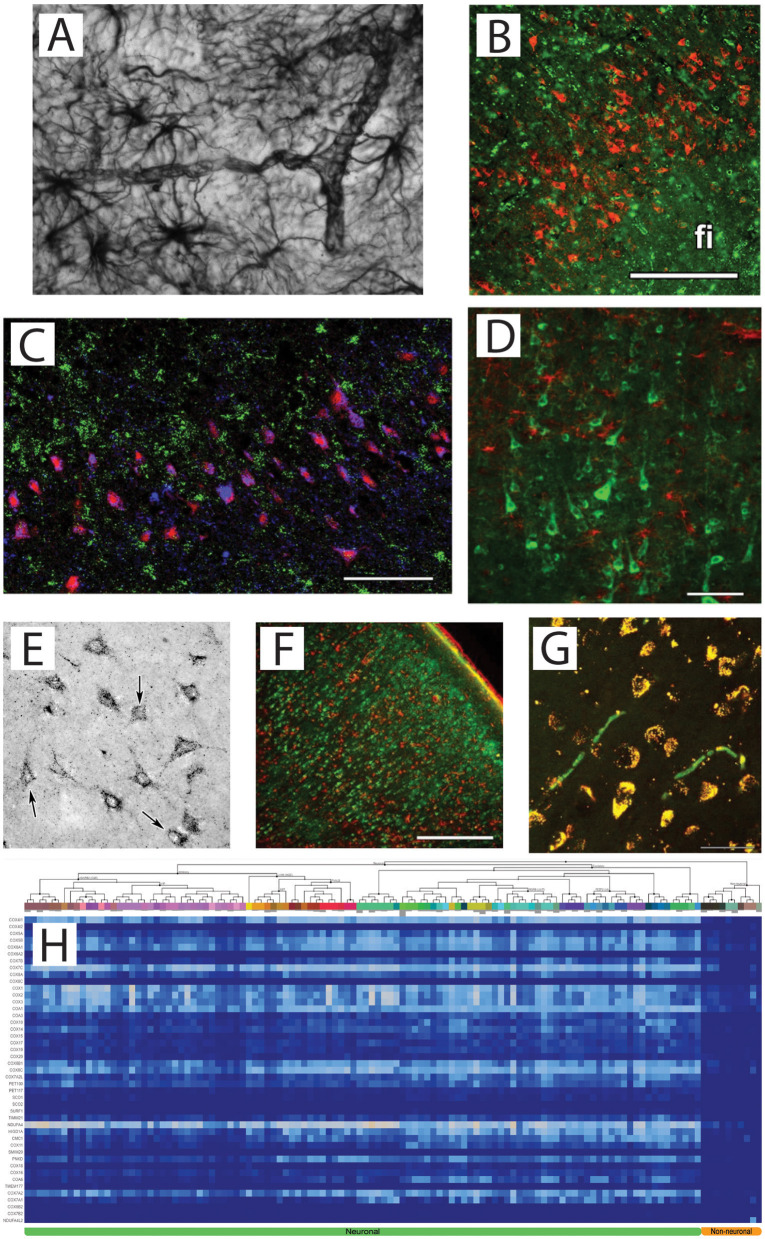
**(A)** GFAP staining (1:200,000) of cortical astrocytes. Their end feet surround the microvasculature so tightly, so that the contour of the vessels are evident. Image courtesy of Dr. Viktoria Vereczki, Department of Anatomy, Semmelweis University. **(B)** Non-astrocytic location of CIV (red) using double immunolabelling with the astrocyte marker S100B (green); fi: fimbria hippocampi. Scale bar = 50 μm. Obtained from Dobolyi et al. (2024). **(C)** Imaging mass cytometry demonstrating CIV (blue), HuB+C + D (red) as neuronal marker, and glial fibrillary acidic protein (green) as a marker of astrocytes. Scale bar = 300 μm. Obtained from Dobolyi et al. (2024). **(D)** A cerebral cortical section double labeled with OGDH isoform 1/2 (green) and the established astrocyte marker glial fibrillary acidic protein (GFAP; red). Scale bar = 250 μm. Obtained from Dobolyi et al. (2020). **(E)** Labeling of MDH2 in the dentate gyrus. Obtained from Dobolyi et al. (2024). **(F)** The distribution of the glial marker S100 (red) is different from that of SUCLA2-positive cells (green) in the human temporal cortex. Scale bar = 500 μm. Obtained from Dobolyi et al. (2015b). **(G)** The mitochondrial localization of SUCLA2 based on its co-localization with the d subunit of the F_0_-F_1_ ATP synthase. Yellow color indicates co-localization of SUCLA2 and F_0_-F_1_ ATP synthase subunit d. An almost complete absence of singly labeled structures can be observed except for the red blood cells. Scale bar = 50 μm. Obtained from Dobolyi et al. (2015b). **(H)** Heatmap of mRNA expression levels in neuronal and non-neuronal cells based on single cell sequencing by the Allen Institute for all subunits participating in Complex IV in human brain (homo) indicated on the y-axis. Queries were made by inputting gene symbols enlisted in MitoCarta 2.0 and curating it to include new data reported in MitoCarta 3.0. Heatmap range is identical to that shown in the last panel of Dataset homo depicting heatmap of homo Pi carriers and dicarboxylate carriers in Dobolyi et al. (2024). On the top part of the x-axis, individual NeuN-positive nuclei (originating from neurons) vs. NeuN-negative (originating from non-neuronal cells including astrocytes), segregated according to the clustering algorithm detailed in https://portal.brain-map.org/atlases-and-data/rnaseq/protocols-human-cortex are depicted. Cell clustering is outlined by the lines branching on the top of the x-axis. Abbreviations are given in https://portal.brain-map.org/atlases-and-data/rnaseq. Non-neuronal elements are shown in the far right of the heatmap, indicated as “Non-neuronal” at the bottom (orange). Neuronal elements are indicated at the bottom of the heatmap in green. Obtained from Dobolyi et al. (2024).

Human brain astrocytes are evolutionarily adapted to exhibit minimal OXPHOS activity for two reasons: (i) Given that astrocytic end feet shape the highly restrictive blood-brain barrier (BBB; see Figure 1A), these cells must avoid depleting O_2_ from the interstitium (the neuronal parenchyma). This is analogous to how low oxygen consumption by endothelial cells ensures sufficient O_2_ reaches even the most distal regions of the vasculature (Groschner et al., 2012). (ii) According to the astrocyte-to-neuron lactate shuttle model (Pellerin and Magistretti, 1994), upon glutamatergic neurotransmission, glycolytically-derived pyruvate in astrocytes is converted into lactate, which is subsequently transferred to neurons. The regeneration of NAD^+^ by lactate dehydrogenase, critical for allowing glycolysis to proceed, is facilitated by the limited expression of key OXPHOS components in human brain astrocytes (Dobolyi et al., 2024). Indeed, complex IV is barely detectable in astrocytes of the adult human neocortex and hippocampal formation when assessed by either immunohistochemistry (Figure 1B) or imaging mass cytometry (Figure 1C; Dobolyi et al., 2024). The absence of complex IV immunoreactivity or enzymatic activity has been documented in brain astrocytes of higher mammals and developing humans since the late 1970s (Wong-Riley et al., 1978; Luo et al., 1989; Hevner and Wong-Riley, 1989, 1991; Wong-Riley, 1989; Hevner and Wong-Riley, 1992; Wong-Riley et al., 1993). Mouse brain astrocytes normally express CIV; however, genetically modified animals lacking this complex specifically in astrocyte mitochondria were viable and showed no evidence of neuronal or glial cell loss, even up to 1 year old (Supplie et al., 2017).

Given the limited OXPHOS capacity of human brain astrocytes, it is unsurprising that major dehydrogenases of the citric acid cycle which supply NADH to CI, are also absent. Specifically, the α-ketoglutarate dehydrogenase complex (KGDHC; Figure 1D; Dobolyi et al., 2020; Ko et al., 2001) and malate dehydrogenase 2 (Figure 1E; Dobolyi et al., 2024) are lacking. Even succinate-CoA ligase is exclusively expressed in human brain neurons (Figure 1F; Dobolyi et al., 2015a,b). Furthermore, essential subunits of mitochondrial F_o_-F_1_ ATP synthase are also predominantly neuronal (Figure 1G; Dobolyi et al., 2015b).

The experimental findings obtained by immunohistochemistry and imaging mass cytometry are strongly corroborated by analyses of transcriptomic data from neuronal and non-neuronal cells extracted from 107 human brains, as deposited in the Allen Institute for Brain Science. These data show that human non-neuronal cells exhibit minimal expression of mRNAs encoding OXPHOS proteins (Dobolyi et al., 2024; see Figure 1H for CIV). This observation differs from the interpretation of the findings by Mosharov et al. (2025), who reported that mRNA abundances of complexes I and IV cluster by voxel rather than by cell type (Figure 3e and Supplementary Data 1). The present critique is confined to cortical gray matter voxels and is not intended to generalize beyond that context. Single-cell transcriptomic datasets nonetheless demonstrate that astrocytic transcripts encoding OXPHOS complexes remain near the detection threshold across all cortical regions, indicating that the voxel-level clustering reported by Mosharov et al. primarily reflects region-specific variation in neuronal expression rather than significant astrocytic contribution. Furthermore, microglia and oligodendrocytes have been shown to express extremely low levels of critical TCA cycle enzymes (Dobolyi et al., 2020, 2015a,b). While their OXPHOS complex levels have not been systematically quantified, these data hint on a similar metabolic limitation beyond astrocytes, corroborated by the finding that essential subunits of mitochondrial F_o_-F_1_ ATP synthase are predominantly neuronal (Figure 1G; Dobolyi et al., 2015b, 2024).

## Refinement of the Mosharov model

Mosharov et al. (2025) calculated the MRC of each brain voxel as follows:


$$TRC=\frac{\left(\sqrt{CI}\right)+\;\sqrt{CII}+\;\sqrt{CIV)}}{3}$$

$$MitoD=\;\frac{\sqrt[{3}]{CS}+\;\sqrt[{3}]{mtDNA)}}{2}$$

$$MRC=\;\frac{TRC}{MitoD}$$

In these formulae, TRC represents tissue respiratory capacity, MitoD mitochondrial density, CI, CII, and CIV correspond to complexes I, II, and IV, respectively, CS stands for citrate synthase, and mtDNA refers to mitochondrial DNA. In gray matter (GM), it is assumed that the ratio of neuronal to non-neuronal cells (primarily astrocytes) is ~1:1 (von Bartheld et al., 2016). Additionally, it is reasonable to presume that neuronal and non-neuronal cells have mitochondria with comparable levels of CS and mtDNA. It cannot be overemphasized that both neuron-glia ratios and neuronal subtypes vary substantially between brain regions, and this would modulate any correction factor on a region-specific basis (Monzel et al., 2025; Green et al., 2023). However, as discussed above, Mosharov et al. (2025) did not account for the fact that only neuronal cells express CIV to an appreciable extent. To the best of my knowledge, full CI and CII enzymatic activities have not been reported in a cell-specific manner in the adult human brain; however, it is safe to conclude that astrocytes cannot perform OXPHOS without CIV (or cytochrome c; Dobolyi et al., 2024).

To account for the fact that only a fraction “F” of cells within a cortical GM voxel are OXPHOS-competent, F·CI, F·CII, and F·CIV are substituted into Mosharov et al. (2025) definition of TRC: TRC_measured = [√(F·CI) + √(F·CII) + √(F·CIV)]/3 = √F · TRC_true. Solving for the true tissue respiratory capacity gives TRC_true = TRC_measured/√F. Because MitoD is unaffected by cell-type composition, the corrected mitochondrial respiratory capacity becomes: MRC_corrected = MRC_measured/√F.

## Discussion

The human brain aerobic energy landscape map created by Mosharov et al. will become a landmark study representing a crucial first step toward achieving their stated goal: to “enable the integration of mitochondrial bioenergetics with macroscopic neuroimaging data, thereby potentially enhancing the specificity of metabolic assessments from PET, BOLD fMRI, CEST MRI, and fMRS by linking them to mitochondrial biology.” Therefore, it is essential that this map be as accurate as possible.

## Data Availability

The original contributions presented in the study are included in the article/supplementary material, further inquiries can be directed to the corresponding author.
